# In vitro testing of silver-containing spacer in periprosthetic infection management

**DOI:** 10.1038/s41598-021-96811-9

**Published:** 2021-08-26

**Authors:** Renate Krassnig, Gloria Hohenberger, Angelika Schwarz, Walter Goessler, Gebhard Feierl, Renate Wildburger, Reinhard Windhager

**Affiliations:** 1 AUVA Rehabilitation Clinic Tobelbad, Dr.-Georg-Neubauer-Straße 6, 8144 Tobelbad, Austria; 2 Department of Orthopedics and Trauma Surgery, Medical University of Graz, Auenbruggerplatz 5, 8036 Graz, Austria; 3 AUVA Trauma Hospital Graz, Göstinger Straße 24, 8020 Graz, Austria; 4 University of Graz, Universitätsplatz 1/I, 8010 Graz, Austria; 5 Diagnostic and Research Institute of Hygiene, Microbiology and Environmental Medicine, Medical University of Graz, Neue Stiftingtalstraße 6, 8010 Graz, Austria; 6 Department of Orthopedics, Medical University of Vienna, Währinger Gürtel 18-20, 1090 Wien, Austria

**Keywords:** Medical research, Preclinical research

## Abstract

Deep infection is a serious complication in endoprosthetic surgery. In correlation to the patient local or systemic compromising factors conservative and surgical proceedings has to be evaluated. Systemic antibiotic therapy is the gold standard in infection management. Implanted silver-coated or silver-containing medical devices have been proven to their antimicrobial effectiveness since the 1990s by several investigators. The outcomes showed that long time implantation could cause damaging of the surrounding tissues, especially of adjacent nerves. The aim of our study was to evaluate the release of silver (I) ions from bone cement mixed with either nanosilver particles (AgNPs), different concentrations of silver sulfate (Ag2SO4) or from pure metallic silver strips. Therefore, we choose two methods: the first, called “static model”, was chosen to evaluate the maximal accumulative concentration of silver (I) ions, with the second, called “dynamic model”, we simulated a continuous reduction of the ions. In an additional test design, the different materials were evaluated for their antimicrobial activity using an agar gel diffusion assay. The outcome showed that neither the addition of 1% (w/w) nanosilver nor 0.1% silver sulfate (w/w) to polymethylmethacrylat bone cement has the ability to release silver (I) ions in a bactericidal/antifungal concentration. However, the results also showed that the addition of 0.5% (w/w) and 1% (w/w) silver sulfate (Ag2SO4) to bone cement is an effective amount of silver for use as a temporary spacer.

## Introduction

Deep periprosthetic infection is a common problem in endoprosthetics, especially in orthopaedic reconstructive treatment. At a percentage of about 2% it is a rare but serious complication^1,2^. Since the early 1990, therapeutic options have been changed rarely. Debridement, lavage, systemic antibiotic therapy, antibiotic loaded bone cement and the change of the medical device, either in a one-way or two-way stage replacement are standard in endoprosthetic infection management^3,4^. In literature, there are in vitro and in vivo studies about the benefit of silver (I) ions activity used on burn wounds or leg ulcers, urinary catheters, heart valves, silver-coated mega-endoprostheses, and central venous catheters^5–12^. Alt et al. reported about the antimicrobial potency of silver (I) ions released from polymethylmethacrylate (PMMA) bone cement loaded with nanosilver particles (AgPNs) in order to eradicate bacterial growth^13,14^. Yamebe et al. reported about the application of silver to prevent biofilm formation on the implanted medical device^15^. Moreover, Flores-Arriaga et al. gave an overview about antimicrobial polymethylmethacrylate with silver nanoparticles for dentistry in their review^16^. In addition to the antimicrobial activity undesirable effects e. g. neuropathy and argyria were described^17–20^. Seokyoung et al. reported about the critical issues of silver nanoparticles (AgNPs) including excessive release of silver (I) ions, severe oxidation, and cytotoxicity^21^.

The aim of this study was to investigate the silver (I) ions release out of a PMMA matrix mixed with silver nanoparticles (AgNPs) and an inorganic Ag (I) salt, silver sulfate (Ag_2_SO_4_), compared to metallic silver in a bactericidal/fungicidal concentration during a maximum implantation time of nine weeks (corresponds to the exposure time of the spacer).

## Materials and methods

Three different kinds of silver products were used. Two of them were mixed with polymethylmethacrylate PMMA bone cement (TRAUMACEM™ V) the third was used in a pure metallic form:

1. Nanosilver with a particle size of 5–50 nm and an active surface of 4 m^2^/g.
2. Silver sulfate in a finely powdered form (particle size: 180 µm ISO standards 565–1972).
3. Pure metallic silver strip.

The PMMA bone cement was mixed with 0.1% (w/w), 0.5% (w/w), 1% (w/w) and 5% (w/w) silver sulfate (Ag2SO4) as well as 1% (w/w) of silver nanoparticles (AgNPs) in the original kit. The liquid bone cement was poured into a mold to in order to form bone cement samples (cylinder) with a thickness of 10, a diameter of 15 mm and a surface of 824.67 mm^2^. The weight of each cylinder was 2 g with a SD of 0.01 g. The pure metallic silver stripe was cut into strips at a length of 50 mm, a breadth of 7 mm and a thickness of 0.2 mm. The weight of the strips was measured by a laboratory scale and represented 0.42 g for each silver strip.

To test the silver ions release from the silver containing bone cement we used two different methods.

The first, called static elution model, allowed the measurement of the maximum concentration of silver ions that may accumulate in the course of 9 weeks. The second one, called dynamic elution model, was used to simulate the reduction of the silver (I) ions.

The samples were placed in polyethylene testing container with a capacity of 60 ml. 50 ml physiological fluid (DMEM—Dulbecco's Modified Eagle Medium) were added into the container. Five samples of each concentration were tested parallel. Then the test container were put into an incubator at a constant temperature of 37 °C. During the incubation period the container were moved on a vibrating table 25 turns per minute to avoid deposition of ions on the wall of the container.

Fluid samples were taken on day 1, 5, 12, 19, 26, 33, 40 and 61. Always performing the same procedure.

Before taking a sample the container were shacked by 80 turns per minute to achieve an even distribution. Then, 1 ml fluid was taken out of each test container and was pipetted into its own eprouvette.

In the static model the taken ml was refilled with fresh DMEM and the tubes were put back to the incubator. In the dynamic model the bone cement samples were taken out from the test container rinsed off with DMEM and put into a new container filled with 50 ml fresh DMEM. The taken samples, all placed into eprouvettes, were stabilized with nitric acid and measured by Inductively Coupled Plasma Mass Spectrometer (ICP-MS).

The tests were carried out in two independent test cycles over a period of 9 weeks.

To investigate the antimicrobial effectiveness of the PMMA bone cement mixed with 0.1% (w/w), 0.5% (w/w), 1% (w/w) and 5% (w/w) silver sulfate (Ag2SO4) as well as 1% (w/w) of silver nanoparticles (AgNPs) as well as the pure metal silver strips, the samples were tested by agar diffusion assay. Therefore, Mueller Hinton agar dishes were inoculated with *Staphylococcus aureus* (ATCC 25923), *Enterococcus faecalis* (ATCC 29212), *Escherichia coli* (ATCC 25922), *Pseudomonas aeruginosa* (ATCC27853), *Bacillus cereus* (ATCC 11778), *Candida albicans* (ATCC 90028) and *Staphylococcus aureus* (ATCC 29213). The Ag-PMMA samples were placed on the dishes. Then, the dishes were placed into an incubation box for 24 h at 37 °C. After the incubation period the diameter of the zone of inhibition of growth was measured and recorded for each sample. The agar diffusion test was carried out in two independent experiment cycles.

### Statistical analysis

All calculations were performed with Statistical Package for Social Sciences (SPSS) 21.0. Concerning descriptive statistics, continuous variables will be presented as mean and standard deviation (SD), median, minimum and maximum, categorical data as frequencies and percentages.

## Results

**Figure 1:**
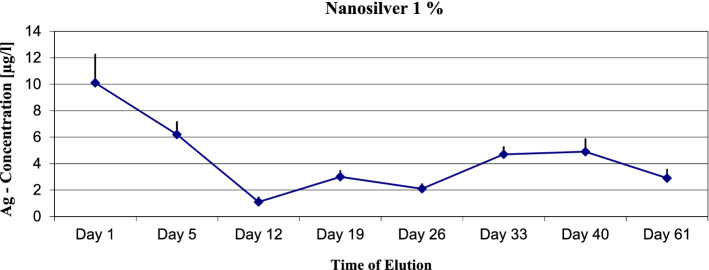
Nanosilver 1%—static elution model—max. Concentration of Ag-Ions 10.1 µg/l.

**Figure 2:**
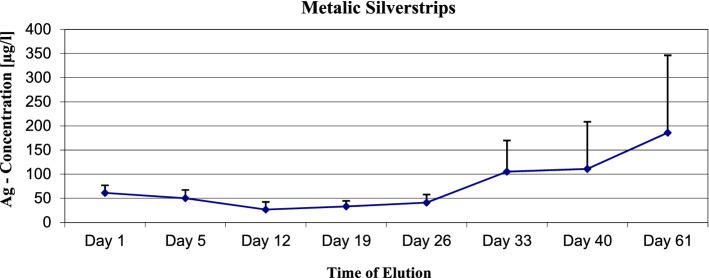
Pure metallic silver strips—static elution model—max. Concentration of silver ions 185.9 µg/l.

**Figure 3:**
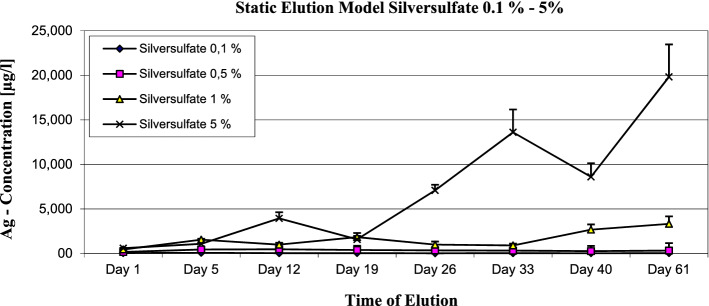
Silversulfate bone cement 0.1 to 5%—static elution model—max. Concentration of silver ions at 0.1% was 70.6 µg/l; at 0.5% was 455.5 µg/l; at 1% was 3321.4 µg/l and at 5% the max. Concentration was 19,826 µg/l.

In the static elution model, the maximum of the expected silver ions concentration was measured. The 1% nanosilver sample showed a maximum concentration of 10.1 µg/l Ag-ions (see Fig. 1). The metallic silver strip sample showed a maximum concentration of 185.9 µg/l Ag ions (see Fig. 2). The silver sulfate bone cement showed a maximum concentration of silver ions of 70.6 µg/l at the 0.1% sample, 455.5 µg/l at the 0.5% sample 3321.4 at the 1% sample and 19,826 µg/l at the 5% sample (see Fig. 3).

The eluted silver ions concentrations from the nanosilver samples and the pure metallic silver strips were lower than the minimal bactericidal concentration. Due to this fact, we did not use these materials at the dynamic elution model.

**Figure 4:**
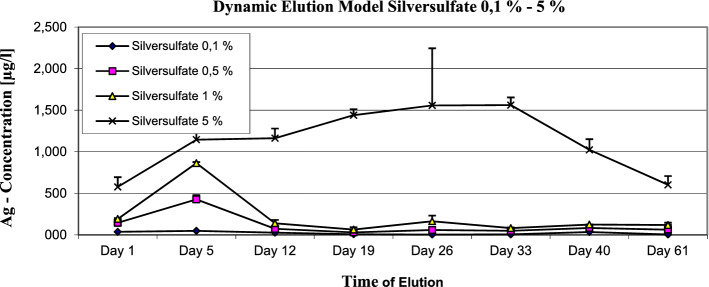
Silver sulfate bone cement 0.1 to 5%—dynamic elution model—max. Concentration of silver ions at 0.1% was 48.57 µg/l; at 0.5% was 427 µg/l; at 1% 863 µg/l and at 5% the max. Concentration was 1561.75 µg/l.

In the dynamic elution testing the silver sulfate containing samples showed maximum concentrations as follow: 0.1% 48.57 µg/l; 0.5% 427 µg/l; 1% 863 µg/l and 5% 1561.75 µg/l (see Fig. 4).

The antimicrobial activity was tested on Mueller Hinton Agar discs. The diameter of the zone of inhibition of growth was measured by using a guide (in mm). Results are listed in Table 1.

**Table 1:** Diameter of zone of inhibition after 24 h of incubation at 37 °C measured in mm.

Microorganism	0.1% Ag_2_SO_4_	0.5% Ag_2_SO_4_	1% Ag_2_SO_4_	5% Ag_2_SO_4_	Blind	Nano-silver	Palacos R + G	Ciprofloxacin
*Staphylococcus aureus* ATCC 25923	0	1	0–2	1–3	0	0	12	27
*Enterococcus faecalis* ATCC 29212	0	0	0–1	0–1	0	0	6	21
*Escherichia coli* ATCC 25922	0	1	1	1–2	0	0	10	34
*Pseudomonas aerug* ATCC 27853	0	1	1–2	1–3	0	0	10–12	34
*Bacillus cereus* ATCC 11778	0	1	1	1–2	0	0	13–14	24
*Candida albicans* ATCC 90028	0–1	0–2	1–2	3–4	0	0	0	0
*Staphylococcus aureus* ATCC 29213	0	0–1	1–2	2	0	0	11	28

Measurements have shown, neither 1% nanosilver nor 0.1% silver sulfate (both percentage values indicating their relation to the molecular weight of silver) added to PMMA nor the use of pure metallic silver strips were able to release silver ions in a bactericide or fungicide concentration^22–24^.

The highest concentration of silver ions release was 455.5 µg/l released by bone cement mixed with 0.5% silver sulfate, 3321.4 µg/l when 1% silver sulfate and 19,826 µg/l when 5% silver sulfate was added.

At the agar diffusion test PMMA mixed with 1% (w/w) nanosilver showed no zone of inhibition. PMMA mixed with Ag2SO4 showed an increasing zone of inhibition with increasing concentration of silver sulfate added to the bone cement (see Table 1).

## Discussion

Periprosthetic joint infection (PJI), particularly candida associated PJI is a rare and difficult to treat complication in endoprosthetic surgery. Candida periprosthetic joint infection constitute a challenge because candida spp. often grow in a biofilm formation adhering to the medical device^25^. Aggravating, candida PJI predominantly effects older patients with various comorbid conditions and perioperative risk factors that limits treatment management^26^.

Since the 1990, silver containing and silver-coated medical devices were investigated due to their antimicrobial activity^9–14^. Recent studies are focused on silver nanoparticles and their bioactivity^16,27^. It was reported that AgNPs are more reactive with a stronger potency and a stronger activity against biofilm formation because of the large active surface area^28^. Abuayyash et al. investigated the efficacy of sacrifical anode containing silver within a bacteria-containing human plasma clot and showed that the antibacterial efficiency of Ag coatings is reduced under tissue-like conditions because of building complexes^29^. For this reason, in surgery it is recommended to avoid hematoma^30^. Souter et al. reported about the variable toxicity of silver ions in cell culture media and reported that DMEM providing a reliable basal media in which to conduct assessments^31^.

Further investigators reported about long-term implanted silver containing devices but literature lacks information concerning silver containing spacers up to now. This study was created to develop a silver-containing, time limited implanted spacer to be effective against microorganisms without damaging potency to the surrounding tissues. Accumulation of silver ions above 1200 µg/l was described to affect human fibroblast growth^32^. Therefore, we investigated silver containing devices with regard to the minimum bactericide/fungicide concentration and the upper limit to negative side effects. Referring to our results, neither nanosilver nor 0.1% silver sulfate admitted to bone cement is able to release silver (I) ions in a bactericidal concentration. The addition of 5% silver sulfate to bone cement showed to pass over the upper limit of benefits. Our results showed that the addition of 0.5% and 1% silver sulfate to bone cement are an effective amount of silver to be used as a temporary spacer.

Deep infection after joint replacement is caused in a majority of cases by biofilm forming bacteria with a significantly high incidence of Staphylococcus aureus and Staphylococcus epidermidis. The building of the biofilm structure includes two steps. In the first step there is a strong adhesive binding to the surface of the implanted device. Secondly, the adherent bacteria begin to produce an extracellular mucus matter consisting exopolysaccharides. Bacteria, living in biofilms are widely metabolism inactive, making antibiotics/antimycotics less effective^26,33–35^.

Since the antimicrobial potency of silver ions was proven, many studies were performed to investigate its benefit to human health; e. g. silver coated films in burn wound surgery, silver containing urinary catheters or vascular grafts, nanosilver containing bone cement, heart valves and furthermore silver coated endoprostheses were tested in vitro and in vivo^5–14^.

In contrast, some investigators capitalized on the adverse effects of silver ions to mammalian cells. Silver (I) ions accumulation higher than 1200 µg/l was described to be cytotoxic^32^. Due to that reason it is very important to know that silver ions might accumulate in the medical device´s surrounding tissues causing complications like agyrosis or the damage of the nerve tissue^17,20^.

The present study reports about silver (I) ions release from different materials. We used PMMA bone cement mixed with either 1% nanosilver or silver sulfate in concentrations from 0.1% up to 5%—all in weight percentage to dry bone cement powder—or pure metallic silver strips. The released amount of silver ions exceeded the minimum inhibitory concentration for the used pathogens from a concentration of 0.5% silver sulfate and more. Nanosilver containing bone cement, the 0.1% silver sulfate containing bone cement and the pure silver strips were not able to release silver ions in an effective amount. Recently, biofilm forming bacteria and candida were investigated with respect to the inhibitory concentration of silver ions on them. Results showed that silver ions at low ppb concentrations are ineffective against cells residing within the biofilm but silver ions are able to destabilize intermolecular adhesion of the biofilm at a concentration of about 50 ppb^25,36^.

## Conclusion

With respect to the activity of silver (I) ions to destabilize biofilm formation and the bactericide/fungicide potency, we argue that the combination of a silver (I) ions releasing spacer and a parenteral antibiotic/antimycotic therapy might be a synergistic trial in a two-stage procedure who patients could benefit from.
